# Germinal Center B Cell Depletion Diminishes CD4^+^ Follicular T Helper Cells in Autoimmune Mice

**DOI:** 10.1371/journal.pone.0102791

**Published:** 2014-08-07

**Authors:** Isharat Yusuf, Jessica Stern, Tom M. McCaughtry, Sandra Gallagher, Hong Sun, Changshou Gao, Thomas Tedder, Gianluca Carlesso, Laura Carter, Ronald Herbst, Yue Wang

**Affiliations:** 1 Department of Respiratory, Inflammation and Autoimmunity Research, MedImmune LLC, Gaithersburg, Maryland, United States of America; 2 Department of Antibody Discovery and Protein Engineering, MedImmune LLC, Gaithersburg, Maryland, United States of America; 3 Department of Immunology, Duke University Medical Center, Durham, North Carolina, United States of America; COCHIN INSTITUTE, Institut National de la Santé et de la Recherche Médicale, France

## Abstract

**Background:**

Continuous support from follicular CD4^+^ T helper (Tfh) cells drives germinal center (GC) responses, which last for several weeks to produce high affinity memory B cells and plasma cells. In autoimmune Sle1 and NZB/W F1 mice, elevated numbers of Tfh cells persist, promoting the expansion of self-reactive B cells. Expansion of circulating Tfh like cells have also been described in several autoimmune diseases. Although, the signals required for Tfh differentiation have now been well described, the mechanisms that sustain the maintenance of fully differentiated Tfh are less understood. Recent data demonstrate a role for GC B cells for Tfh maintenance after protein immunization.

**Methods and Finding:**

Given the pathogenic role Tfh play in autoimmune disease, we explored whether B cells are required for maintenance of autoreactive Tfh. Our data suggest that the number of mature autoreactive Tfh cells is controlled by GC B cells. Depletion of B cells in Sle1 autoimmune mice leads to a dramatic reduction in Tfh cells. In NZB/W F1 autoimmune mice, similar to the SRBC immunization model, GC B cells support the maintenance of mature Tfh, which is dependent mainly on ICOS. The CD28-associated pathway is dispensable for Tfh maintenance in SRBC immunized mice, but is required in the spontaneous NZB/W F1 model.

**Conclusion:**

These data suggest that mature Tfh cells require signals from GC B cells to sustain their optimal numbers and function in both autoimmune and immunization models. Thus, immunotherapies targeting B cells in autoimmune disease may affect pathogenic Tfh cells.

## Introduction

Germinal centers (GC) are the prominent places for generation of self-reactive B cells in autoimmune diseases and GC responses are driven mainly by CD4^+^ T-helper cells confined within B cell follicles called T follicular helper (Tfh) cells [1]–[11]. Throughout the course of GC responses, Tfh cells persistently provide an array of signals to GC B cells, such as CD40 ligand (CD40L), interleukin (IL)-21 and IL-4, which in combination support GC B cell proliferation, somatic hypermutation, immunoglobulin class switching, and eventual differentiation into memory B cells and plasma cells [4],[12]–[14]. Increased numbers of Tfh cells and/or dysregulated Tfh function contribute to the development of autoimmune phenotype in multiple autoimmune mouse models and expansion of Tfh-like cells have been reported in the peripheral blood from patients with Systemic lupus erythematosus (SLE), primary Sjögren's syndrome, rheumatoid arthritis and myasthenia gravis patients [2], [3], [15]–[32]. Together, these findings suggest that Tfh cells are promising therapeutic targets in autoimmune patients.

Recent studies using immunization or infection models have shed light on the pathways leading to the development of Tfh cells in these models [4], [33]. First, Tfh cells require Bcl-6 for their development and proper function [34]–[36]. Second, antigen presenting cells (APCs) play important roles for Tfh development, with dendritic cells and B cells working in tandem at different stages of Tfh differentiation [37]–[39]. Third, several signaling pathways including CD28, ICOS, and SAP have been shown to be critical for Tfh differentiation [4]. Finally, in an Ovalbumin immunization model, the maintenance of the Tfh cells throughout the course of GC responses was dependent on persistent antigen presentation and ICOS-ICOSL signals provided by GC B cells [40]. However, it was also reported in other mouse models that Tfh cells can be induced and maintained for long period of time in the absence of B cells [41].

Less is known about mechanisms which support the maintenance of Tfh in autoimmune diseases and few therapies that can directly target Tfh cells have been identified. Given the role of B cells in Tfh differentiation and maintenance described in immunization models, we explored whether in mouse models of autoimmunity, signals provided by GC B cells are required to maintain the Tfh phenotype [4], [33], [40]. This is a clinically relevant question because multiple models of autoimmune-prone mice have been reported to have presence of spontaneous GCs at the onset of disease manifestations [1]. In addition, several therapeutic approaches have been developed to block T-dependent B cell responses; however, whether these therapies can diminish number of Tfh cells are not clear [42], [43]. Finally, it is not obvious whether the mechanisms of B cell-dependent differentiation and maintenance described in immunization models would be similar in spontaneous models of autoimmunity where Tfh expansion could result from T cell-intrinsic B cell-independent mechanisms [4], [33], [40].

Here we found that in autoimmune mice without immunization or obvious infections, numbers of Tfh cells are significantly higher and accumulate over time when compared to syngeneic age-matched and otherwise healthy mice. The current study was undertaken to identify the signals that sustain mature Tfh cells in autoimmune and immunization settings. We hypothesized that a GC-dependent ‘feed forward loop’ is responsible for accumulation of Tfh in pathological settings seen in spontaneous models of lupus-prone mice. Indeed, therapeutic depletion of total B cells or specifically GC B cells in autoimmune-prone models, such as Sle1.hCD19 Tg and NZB/W F1 mice, caused a significant reduction in numbers of Tfh cells. Similarly and consistent with what has been described previously with protein immunizations, disruption of GC B cells in BALB/c mice immunized with Sheep Red Blood Cells (SRBC) led to an accelerated reduction of Tfh numbers and diminished IL-21 production [40]; demonstrating that in both settings of spontaneous autoimmunity and immunization with exogenous antigen, mature Tfh depend on GC B cells for optional maintenance. Additionally, our data show that the maintenance of mature Tfh in autoimmune mice appears to be dependent on the ICOS-ICOSL and CD28 signaling pathways. In contrast, the latter pathway is not required for Tfh maintenance in SRBC immunized mice, highlighting a key mechanistic difference between autoreactive Tfh and Tfh generated by immunization with an exogenous antigen. These results provide evidence that GC B cells control the maintenance of mature effector Tfh cells and this GC B cell-dependent mechanism can lead to the accumulation of pathogenic Tfh in autoimmune disease models. These findings also contribute to a better understanding of B cell-targeted therapies and therapeutic approaches that aim to disrupt Tfh-B cell communication and co-stimulation.

## Materials and Methods

### Ethics

The study was carried out in strict accordance with the recommendations in the Guide for the Care and Use of Laboratory Animals of the National Research Council. The animal experiments were conducted at MedImmune in accordance with protocols (MI-12-0018 and MI-12-0001) approved by the Institutional Animal Care and Use Committee of MedImmune Lab Animal Research.

### Mice

C57BL/6, BALB/c and NZB/W F1 mice were purchased from the Jackson Laboratory and mice between ages of 2 to 8 months were used for experiments. Sle1 and human CD19 transgenic (hCD19Tg) mice, both on a C57Bl/6 background were described previously and here were bred to create mice homozygous for Sle1 and hemizygous for the hCD19 transgene; these are referred to as SLE1-hCD19Tg mice [44]. Sle1 was identified using microsatellite primers, and zygosity of the hCD19 transgene was determined by flow cytometry using an anti-hCD19 antibody. Both male and female Sle1-hCD19Tg mice, between 6 to 12-month old, were used for studies. All mice were bred at Jackson Laboratory and housed in the animal research facility at MedImmune. All animals were euthanized via CO2 followed by cardiac puncture or cervical dislocation under anesthesia.

### Antibodies and fusion proteins

Anti-CD40L TM MAb was engineered and produced at MedImmune. The variable region sequences from a hamster IgG anti-murine CD40L (clone MR1) were synthesized from published sequence [45] then cloned in-frame with sequences of human IgG1 constant region with 3 mutations L234F/L235E/P331S (hIgG1 TM). These mutations in IgG1 Fc have been shown to cause a profound decrease in the binding to Fcγ receptors and fail to induce MAb dependent cytotoxicity [46], [47]. Anti-ICOS-TM antagonist MAb was made by linking the variable region sequences from a rat IgG2a anti-murine ICOS (clone JMAb-51, provided by Central Pharmaceutical Research Institute of Japan Tobacco) with sequences of human IgG1-TM. Vectors containing full-length sequences of MR1-hIgG1 TM and J51-hIgG1 TM were transfected into 293 cells and antibodies were purified from supernatant. These MAbs are referred to as anti-CD40L-TM and anti-ICOS-TM. The binding properties were identical between the parental antibodies and the engineered antibodies with human IgG1-TM.

Both anti-human CD19 (clone MEDI551) and anti-murine CD20 (clone MB20-11) Ab have been used before for immuno-depletion of B cells in mouse models [46], [48]. LTβR-Ig and CTLA4-Ig were produced following published method [49], [50]. Isotype control MAbs were purchased from BioXCell.

### In vivo treatment and immunization

MAb and fusion proteins were prepared in PBS, with low endotoxin (<0.1 EU/mg) and injected intravenously. To induce GC reaction, naïve BALB/c mice were immunized with 0.2 ml of SRBC (Colorado Serum Company), by intraperitoneal injection after withdrawing directly from bottle.

### Flow Cytometric Analysis

Single-cell suspensions were prepared from spleens by standard gentle mechanical disruption. Antibodies for surface staining, anti-B220 (RA3-6B2), anti-CD44 (IM7), anti-SLAM (TC15-12F12.2), anti-IgD (11-26c.2a) were purchased from Biolegend. Anti-CD95/Fas (Jo2), anti-T-and B-cell activation antigen (GL7), anti-CD19 (ID3), anti-CD4 (RM4-5) were purchased from BD Biosciences. Anti-PD1 (J43) and anti-IgM (Il/41) were purchased from eBioscience. CXCR5 staining was done using either purified anti-CXCR5 or biotin labeled anti-CXCR5 (BD Biosciences) and performed as previously described [51]. Anti-CD8a (5H10) and Alexa Fluor 647 labeled streptavidin was purchased from Life Technologies. GC were detected with PNA-FITC (Vector Laboratory) and all surface stains included viability dye, 7-AAD (eBioscience). For intracellular staining, splenocytes were first stained with surface makers followed by staining with live/dead fixable cell staining from Life Technologies. Cells were then fixed and permeabilized using Foxp3 Staining Buffer set purchased from eBioscience following manufacturer's protocol. Cells were stained for intracellular proteins with anti-Bcl-6 (K112-91, BD Biosciences) and anti-Ki67 (B56, BD Biosciences) and Foxp3 (FJK-16s, eBioscience).

### Immunofluorescence Microscopy and Immunohistochemical Analysis

Spleens from SLE1.hCD19Tg and immunized BALB/c mice were harvested, embedded in Frozen Tissue Media (Tissue-Tek O.C.T. Compound, Sakura Finetek) and snap frozen in liquid nitrogen. Four micrometer-thick frozen sections were fixed in acetone for 15 min and dried in air for 30 min. The sections were blocked with PBS containing 3% fetal bovine serum for 30 min at room temperature and were then stained for 30 min at room temperature with cocktails of antibodies and PNA. The following conjugations with Alexa fluor dyes (Life Technology) were performed according to the manufacturer's instructions: PNA (Vector Laboratory) to Alexa 488; anti-PD1 (RMP1-30, eBioscience) and anti-CD35 (8C12, BD Bioscience) to Alexa 555 and anti-CD4 (GK1.5, BioXcell) to Alexa 647. Anti-IgD eFluor 450 was purchased from eBioscience. Sections were mounted in Fluormount G (Southern Biotechnology Associates) and viewed with a Leica TCS SP5 microscope. False colors were used to increase contrast. Histology experiments were repeated on at least three mice, and representative staining patterns are shown.

### RNA expression

BALB/c mice were immunized with SRBC, followed by treatment with either anti-CD20+anti-CD40L-TM or isotype controls at 9 days post immunization. Eight days after antibody treatment, CD4 T cells were purified by negative selection using magnetic beads (Miltenyi). Enriched cells were sorted on the basis of CD44^high^CD62L^low^ expression using a FACSAria (BD Biosciences). Approximately 1×10^6^ cells were sorted from each group in duplicate (each replicate consisted of 5 pooled spleens). RNA was isolated using RNAeasy Plus Mini isolation kit from Qiagen according to manufacturer's protocol. cDNA synthesis was performed using SuperScript III Reverse Transcriptase (Life Technologies) with random hexamer primed reactions.

Gene expression was determined by running reactions in triplicate using BioMark Dynamic Array (Fluidigm Corporation) microfluidics system, which has a tight correlation with conventional RT-PCR. Validated Taqman probes were purchased from Applied Biosystems for IL-21 (Mm00517640_m1), Bcl-6 (Mm00477633_m1) and 18S (Hs99999901_s1). Average Ct values were used to determine sensitivity and specificity of the designed probes. Cts above 20 were excluded from the calculation. Delta-delta Cts (ΔΔCt) were calculated using the mean of the reference gene (18S) and a calibrator sample and were converted to fold expression change by the following formula: 2^−ΔΔCt^ [52].

### Statistical Analysis

Statistical tests were performed using Prism 5.0 (GraphPad). Unless specifically stated, p-values were calculated using two-tailed unpaired Student's t tests with 95% confidence interval. Error bars depict the standard error of the mean (SEM) unless otherwise indicated.

## Results

### Tfh cells are persistent in spontaneous autoimmune-prone mice and in antigen-challenged wild-type mice

**Figure 1:**
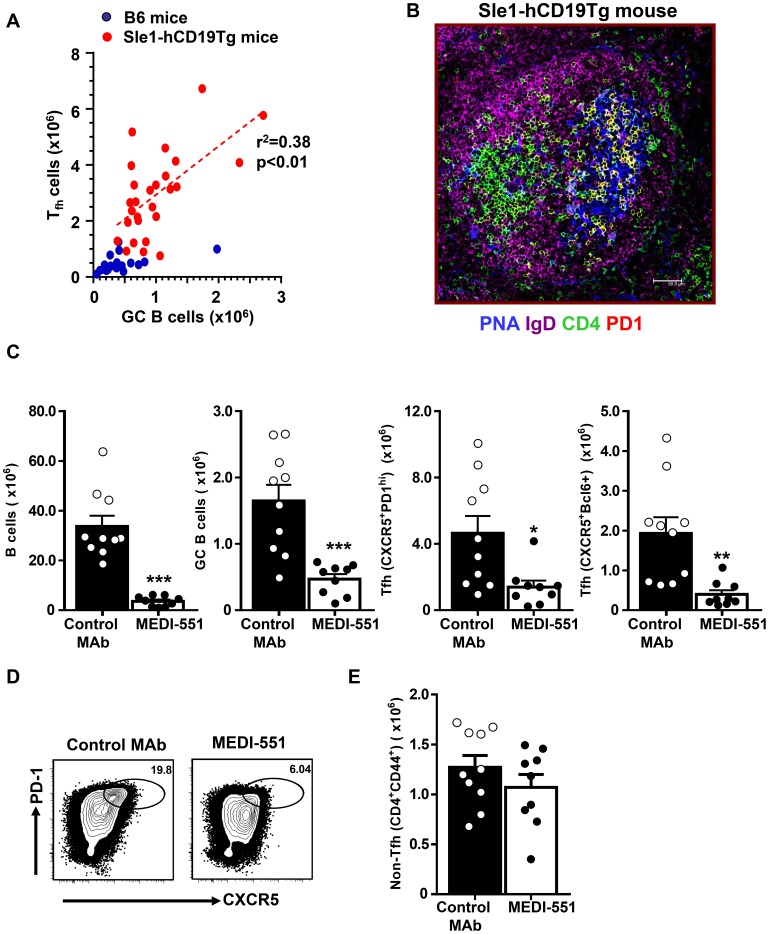
Depletion of splenic B cells in Sle1-hCD19Tg mice leads to a significant reduction of Tfh cells. (A) Spleens were harvested from 5 to 12-month old Sle1-hCD19Tg mice and age-matched C57BL/6 (B6) control mice. Numbers of GC B cells (B220^+^CD19^+^PNA^+^FAS^high^IgD^low^) and Tfh cells (CD4^+^CXCR5^+^PD1^high^) were enumerated by flow cytometry analysis and plotted in the graph. Each dot represents a single mouse of indicated genotype. (B) Immunofluorescence images of frozen spleen sections from a 10-month old Sle1-hCD19Tg mice. Cryosection are stained with PNA (for GC B cells, blue), IgD (naïve B cells, magenta), CD4 (green), and PD1 (red). Tfh cells are CD4 and PD1 double positive and therefore appear yellow. Majority of Tfh cells colocalize with PNA^+^ GC B cells. (C–E) Sle1-hCD19Tg mice were treated with a single dose of an anti-CD19 depleting MAb (MEDI-551) or control MAb (10 mg mg/kg) and spleen cells were collected for FACS analysis seven days after MAb administration. (C) Bar graphs show the number of B cells (B220^+^CD19^+^), GC B cells (PNA^+^FAS^high^IgD^low^) and Tfh cells (CXCR5^+^PD1^high^ or CXCR5^+^Bcl-6^+^). Bars represent the mean value for each group and error bars are standard error of the mean. (D) Representative FACS contour plots showing CXCR5 versus PD1 staining gated on CD4^+^CD44^high^ cells from Sle1-hCD19Tg mice with indicated treatment. Gates have been drawn around the populations representing Tfh cells. (E) Bar graph showing the number of non-Tfh cells within CD4^+^CD44^high^ effector and memory T cell gate (CD44^high^CXCR5^−^PD1^low/−^). *** P<0.001 **, P<0.01 *, P<0.05. Data in (C–E) are pooled from two independent experiments.

In autoimmune-prone mouse strains such as Sle1 and NZB/W F1, aged mice spontaneously develop auto-antibodies and have increased numbers of splenic GC B cells and Tfh cells (Figure 1A–B and Figure S1A-B) [53]–[56]. In both autoimmune backgrounds, the numbers of GC B cells and Tfh steadily increase over time as the mice age (Figure S1A–B). Furthermore, the number of Tfh cells (CD4^+^CD44^high^CXCR5^+^PD1^high^) positively correlated with the number of GC B cells (B220^+^CD19^+^PNA^+^Fas^high^IgD^low^) in spleens from 5–12 month old Sle1 mice transgenic for human CD19 (Sle1-hCD19Tg), in comparison with unimmunized age- and sex-matched C57Bl/6 (B6) wild-type mice (Figure 1A and Figure S1B). In the B6 and hCD19Tg spleens both Tfh and GC B cells were consistently scarce (both <0.5×10^6^). In comparison, spleens from age-matched 5–12 month old Sle1 and Sle1-hCD19Tg mice contained on average 6-fold more splenic Tfh cells and 2-fold more GC B cells (means of Tfh 3×10^6^, GC B cells 1×10^6^) (Figure S1A). Majority of splenic Tfh cells from the Sle1.hCD19Tg and NZB/W F1 mice express Bcl-6 and GL-7 and co-localize with GC B cells as shown by CD4^+^PD1^+^ double staining in yellow (Figure 1B and Figure S1E). Less than 10% CXCR5^+^PD1^high^ CD4 T cells express the proliferation marker Ki67 or Foxp3, similar to what has been reported for classical mature Tfh cells induced by immunization (Figure S1E) [4].

We then examined the kinetics of Tfh development in correlation with that of GC B cells in an immunization model of T-dependent B cell response with the goal of identifying the time point at which mature Tfh are present. BALB/c mice were immunized with SRBC and the kinetics of the ensuing GC response was followed as previously described [57]. GC B cells and Tfh cells were identified again by surface marker expression (B220^+^CD19^+^PNA^+^Fas^high^IgD^low^ for GC B cells and CD4^+^CD44^high^CXCR5^+^PD1^high^ for Tfh cells) as well as by intracellular staining for Bcl-6, which is expressed in both GC B cells and Tfh cells [4], [58]. In naive BALB/c mice, average numbers of GC B cells in the spleen as detected by flow cytometry, were <5×10^4^, and the numbers of Tfh cells were <1×10^4^ (Figure S2A–B). Within the first week following immunization with SRBC, Tfh and GC B cells increase and reach peak values between days 7 and 9 (time point when mature Tfh are present) then slowly decreasing over time (Figure S2A–B). On Day 30, the average numbers of Tfh and GC B cells were 6.6×10^4^ and 3.8×10^5^ respectively, representing 39% and 13% of peak values, but 6- and 7-fold higher than those in naïve mice (Figure S2A–B). Histological analysis demonstrated a robust GC response at day 9, with smaller, but clearly detectable, GC between days 14 and 30 post immunization. The majority of PD1^high^CD4^+^ Tfh cells were located within GC at all time points as indicated by the CD4^+^ PD1^+^ double positive yellow cells (Figure S2D). These results indicate that Tfh, similar to GC B cells, can persist for at least one month following immunization with SRBC.

PD1^high^CXCR5^+^CD4^+^cells have previously been reported which include GL7^+^ Tfh cells that are concentrated inside the GC and identify the most mature Tfh subset and most recently, Foxp3^+^ follicular regulatory (Tfr) cells., and Ki67^+^ proliferating Tfh [51], [59], [60]. Ki67^+^ proliferating Tfh cells were found to peak 5 days after SRBC immunization and then gradually decreased with time, and similar trends were noted with the other Tfh subsets (Figure S2C). GL7^+^ marked a stable subset of Tfh cells, representing 50–80% of total Tfh cells at all time points and again peaking between days 7 and 9 post immunization (Figure S2C). The numbers of Foxp3^+^ Tfr cells were relatively low throughout the GC response (Figure S2C). In general, after day 9 GL7^+^ and Ki67+ Tfh and Foxp3+ Tfr cells follow a similar kinetic pattern as the total Tfh cells suggesting that mature Tfh are present by this time point (Figure S2A–D). In SRBC immunized BALB/c mice, the numbers of Tfh cells were several-fold lower than those of GC B cells throughout the course of the response (Figure S2A–B), consistent with the idea that T cell help is a limiting factor in the physiological GC response [4], [61]. In comparison, numbers of Tfh cells are higher than GC B cells in most aged Sle1.hCD19Tg mice analyzed in this study (Figure 1A). Therefore aged Sle1 and Sle.hCD19Tg mice have excess numbers of Tfh, which may contribute to the development of the autoimmune phenotype.

### Depletion of B cells leads to a reduction of Tfh cells in Sle1 and NZB/W F1 mice

Tfh provide critical signals to generate humoral responses and as shown elsewhere and above, B cells and Tfh are in close proximity during GC reactions, therefore we hypothesized that B cells may provide signals back to Tfh cells that contribute to their persistence [4]. This hypothesis was tested by depleting B cells in 9–12 month old Sle1-hCD19Tg in which increased numbers of Tfh cells and GC B cells are consistently noted. These mice were treated with a single injection (10 mg/kg) of isotype control MAb or MEDI-551, an anti-human CD19 MAb previously shown to effectively deplete most B cells in hCD19Tg mice, and splenocytes were recovered and analyzed by flow cytometry on day 7 post treatment [46], [62]. Compared to mice that received isotype control MAb, mice treated with MEDI-551 had greater than 90% reduction of total splenic B cell numbers, including a significant (p<0.001) decrease of splenic GC B cells that were reduced by 70% (Figure 1C). Strikingly, MEDI-551 treatment also resulted in a 70–80% reduction of splenic Tfh cells (p<0.05) whereas numbers of CD44^high^CXCR5^−^PD1^low/−^ non-Tfh CD4 memory and effector T cells were comparable between MEDI-551 and control MAb treated mice (Figure 1C–E). Since Tfh cells from Sle1-hCD19Tg mice do not express transgenic human CD19 (data not shown), the observed effect of MEDI-551 treatment on Tfh cells is likely indirect and resultant of loss of B cells. These results support the hypothesis that persistence of Tfh cells in the Sle1-hCD19Tg mice is likely dependent on the presence of sufficient numbers of B cells. Prolonged treatment (4 weeks or longer) of MEDI-551 led to a reduction of not only Tfh but also total CD44hiCD4+ T cells, similar to the effect of long term treatment of anti-CD20 MAb in autoimmune mice [63], [64].

We then extended our studies to NZB/W F1, another autoimmune mouse model, to confirm if Tfh maintenance is dependent on B cells, and particularly on GC B cells, in mice that develop autoimmune manifestations. NZB/W F1 mice were followed monthly, starting at 2 months of age for the development of GC and Tfh cells in spleens by flow cytometry. By 5 months of age, mice were found to have elevated numbers of Tfh and GC B cells (both>1×10^5^) (Figure S1B). To assess the role of B cells and particular GC B cells in Tfh persistence, five-month old NZB/W F1 mice were treated with anti-CD20 MAb (MB20-11) [64] to deplete B cells and/or anti-CD40L-TM MAb (MR-1) to inhibit CD40-CD40L interaction and specifically eliminate GC B cells [65]–[70]. In the NZB/W F1 model, mice develop proteinuria on average by 6 months of age in our facility, which is quickly followed by morbidity. Therefore, in this model, mice between the ages of 5 to 6 months were used for MAb treatment studies; which could explain the lower numbers of GC B cells and Tfh compared to the Sle1 model where mice between the ages of 9 to 12 months of age were used (Figure S1A and B). To mitigate antibody-dependent cell-mediated cytotoxicity [71], a triple mutant “TM” engineered MR1 MAb was used to remove Fc effector function but retain the CD40L blockade function of MR1 [47].

**Figure 2:**
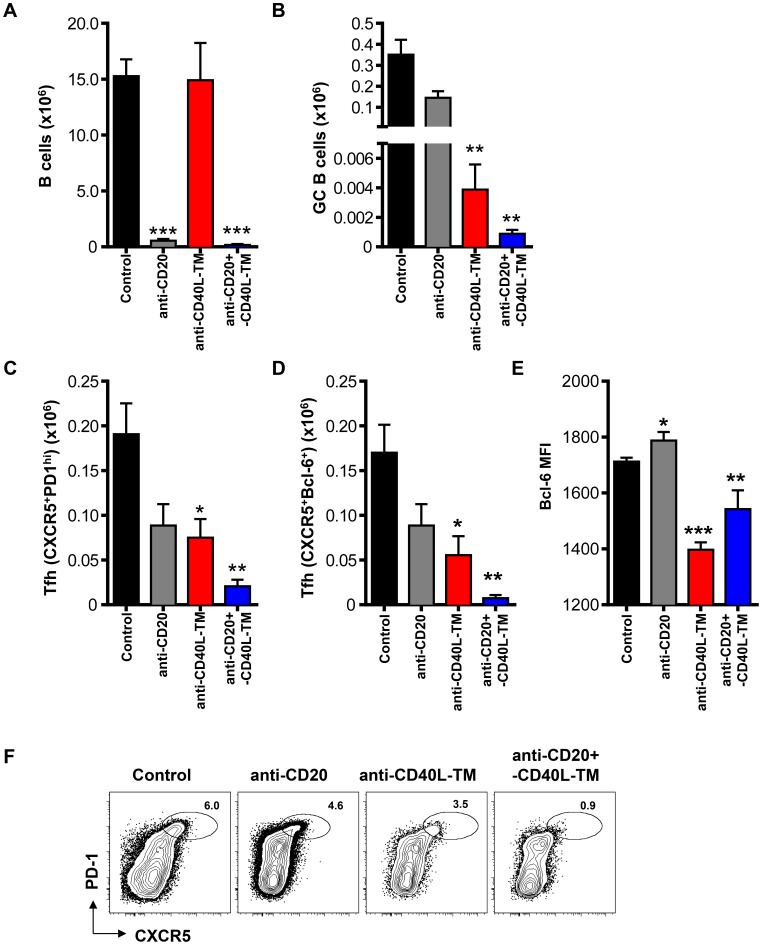
Reduction of GC B cells decreases the number of Tfh cells in NZB/W F1 mice. (A–F) Female NZB/W F1 mice at 5–6 months of age were treated with anti-CD20 (1 mg mg/mouse), anti-CD40L-TM (1 mg mg/mouse), combination of anti-CD20 and CD40L-TM (1 mg each mg each/mouse) or isotype control antibodies (1 mg each mg each/mouse) intravenously on days 0 and 2. Mice were sacrificed and splenocytes were analyzed by FACS at day 7 post first antibody dosing. Bar graph shows numbers per spleen for (A) B cells (CD19^+^B220^+^), (B) GC B cells (PNA^+^FAS^high^IgD^low^), (C) Tfh cells (CD4^+^CD44^high^CXCR5^+^PD1^high^) and (D) Tfh cells (CD4^+^CD44^high^CXCR5^+^Bcl-6^+^). (E) Bar graph shows the mean fluorescence intensity of Bcl-6 for Tfh (CD4^+^CD44^high^CXCR5^+^Bcl-6^+^) cells. ***P<0.001 **, P<0.01 *, P<0.05. Bars represent the mean value for each group and error bars are standard error of the mean. Data are pooled from three independent experiments. (F) Representative FACS contour plots showing PD1 versus CXCR5 staining gated on CD4^+^CD44^high^ cells from mice with the indicated treatment 7 days after MAb dosing. Oval gates show the Tfh (CXCR5^+^PD1^high^) subsets.

Treatment with anti-CD20 MAb alone was sufficient to deplete >95% of total splenic B cells compared to control MAb, but only resulted in a ∼60% reduction of GC B cells, (Figure 2 A–B). In contrast, anti-CD40L-TM MAb, as previously described, did not affect the number of total splenic B cells but instead effectively reduced the number of GC B cells by more than 95% (Figure 2 A–B; p<0.01) [65]–[67]. A combined treatment of anti-CD20 plus anti-CD40L-TM led to >95% reduction in total B cells including a dramatic (>99.5%) decrease in GC B cells compared to mice treated with control antibodies (Figure 2 A–B; p<0.01). Interestingly, the reduction of splenic Tfh cells paralleled the extent of reduction of GC B cells: Tfh cells were reduced by 48% in anti-CD20 MAb-treated mice (p>0.05), 67% in anti-CD40L-TM MAb-treated mice and 96% in anti-CD20 plus anti-CD40L-TM MAb-treated mice (Figure 2 C–D, F; p<0.05 and p<0.01 respectively). Additionally, even though few Bcl-6^+^CXCR5^+^ Tfh cells were still detectable in anti-CD40L-TM or anti-CD20 plus anti-CD40L-TM-treated mice, the mean fluorescence intensity of Bcl-6 on Tfh cells was significantly reduced (Figure 2E). Collectively our data suggest a requirement of B cells, particularly GC B cells in supporting Tfh maintenance in autoimmune mice.

### Depletion of GC B cells leads to a reduction of Tfh cells in SRBC immunized BALB/c mice

We next investigated whether B cells are required for maintenance of Tfh cells during a response to exogenous antigen. Here BALB/c mice were immunized with SRBC, then depleted of B cells by treatment of anti-CD20 MAb at the peak of the GC response (Day 9; Figure S3A). The treatment led to a >90% reduction in total splenic B cell numbers. But consistent with a previous report, anti-CD20 MAb treatment was ineffective in reducing GC B cells compared to PBS-treated mice (Figure S3B–C) [40]. Importantly, no loss of Tfh was noted in these mice in which GC B cells were not depleted (Figure S3D). These data in combination with the early study suggest that while Tfh cells appear to be sensitive to the loss of GC B cells, they do not appear to be affected by the loss non-GC B cells [40].

**Figure 3:**
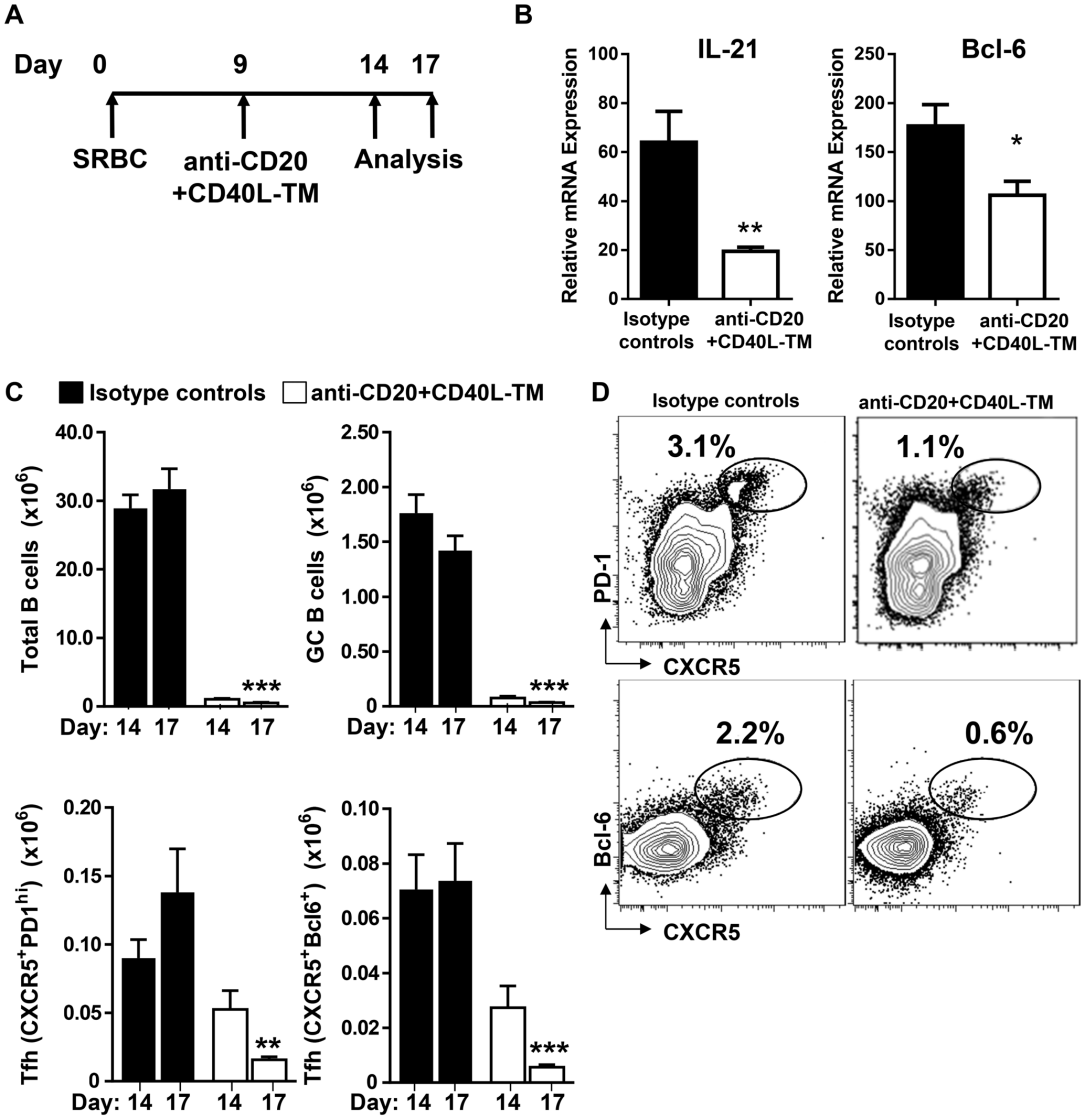
Depletion of B cells in SRBC immunized mice with anti-CD20 plus -CD40L-TM treatment leads to accelerated loss of mature Tfh cells. (A) Diagrammatic representation of experimental protocol. BALB/c mice were immunized with SRBC and treated with either anti-CD20 (0.25 mg mg/mouse) + anti-CD40L-TM (0.40 mg mg/mouse) or control antibodies at day 9 post treatment. Mice were sacrificed on days 5 and 8 post antibody treatment (14 and 17 days post immunization). (B) Relative expression of IL-21 and Bcl-6 in FACS sorted CD4^+^CD44^high^CD62L^low^ cells from mice treated with either anti-CD20+anti-CD40L-TM or control antibodies. Data are normalized with expression of housekeeping gene 18S. (C) Graphs show number of total B cells (B220^+^CD19^+^), GC B cells (PNA^+^FAS^high^IgD^low^), Tfh cells (CXCR5^+^PD1^high^ and CXCR5^+^Bcl-6^+^ cells) gated on CD4^+^CD44^high^ T cells. ***P<0.001 **, P<0.01 *, P<0.05. Data are pooled from two independent experiments. Bars represent the mean value for each group and error bars are standard error of the mean. (D) FACS contour plots show CXCR5 versus PD1 and CXCR5 versus Bcl-6 after gated on CD4^+^CD44^high^ T cells from mice with the indicated treatment. Oval gates show the Tfh subsets.

We then explored whether treatment with both anti-CD20 plus anti-CD40L-TM MAbs which eliminates both GC and non-GC B cells, would affect the maintenance of Tfh cell numbers during an ongoing SRBC response (Figure 3). SRBC-immunized BALB/c mice were dosed with anti-CD20 MAb (0.25 mg) plus anti-CD40L-TM (0.4 mg) at day 9 following immunization (Figure 3A) and the expression of the Tfh associated genes IL-21 and Bcl-6 in CD4+CD44highCD62Llow cells from anti-CD20 plus anti-CD40L-TM treated mice were significantly reduced (Figure 3B). The numbers of Tfh cells and GC B cells in the spleen were quantified by flow cytometry on days 14 and 17 post immunization. Treatment with the combination of anti-CD20 and anti-CD40L-TM MAbs led to a >95% reduction of total B cells, including a profound reduction in GC B cells (>90%) (Figure 3C; p<0.001 and p<0.01 respectively), consistent with the results in NZB/W F1 mice (Figure 2). Importantly, the combination treatment that resulted in loss of both GC and non-GC B cells also resulted in a great reduction of Tfh numbers by 90% compared to those in mice treated with isotype controls (Figure 3C–D; p<0.001).

**Figure 4:**
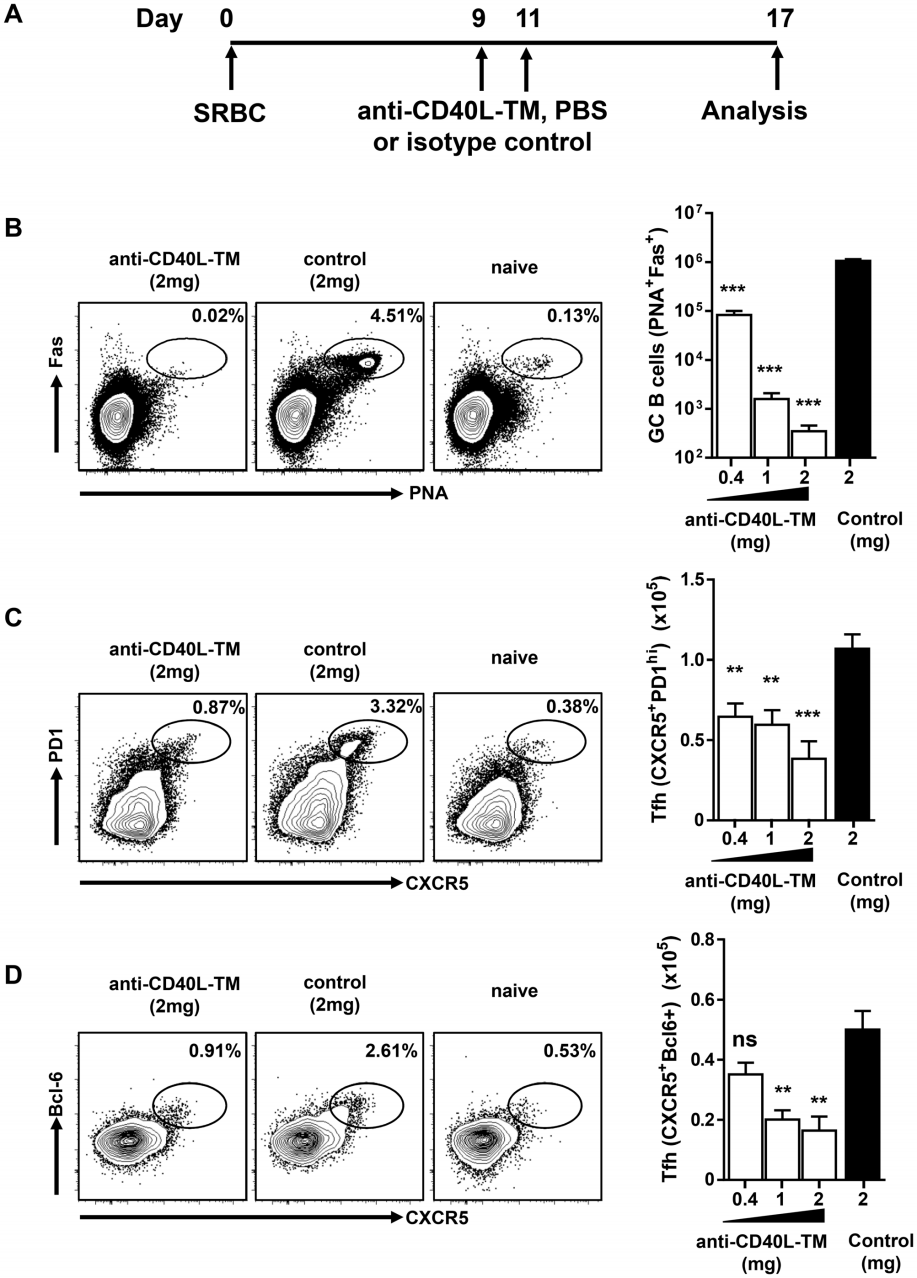
Elimination of GC B cells in established GC reduces the number of mature Tfh cells in SRBC immunized BALB/c mice. (A) A schematic view of SRBC immunization and anti-CD40L-TM treatment protocol: a cohort of BALB/c naïve mice were immunized with SRBC at day 0 and were treated on days 9 and 11 with indicated amounts of either anti-CD40L-TM or isotype control. (B) FACS contour plots show the gate and percentage of GC (PNA^+^FAS^high^) cells after gated on live B cells, from mice treated with MAbs or naïve mice. Bar graph shows number of splenic GC B cells in mice treated with increasing doses of anti-CD40L-TM or isotype control. (C–D) Mice are same as in (B). FACS contour plots show the gate and percentage of Tfh cells (CXCR5^+^PD1^high^) in (C) and (CXCR5^+^Bcl-6^+^) in (D) after gating on CD4^+^CD44^high^ cells and bar graphs show numbers of Tfh cells per spleen. ***P<0.001 **, P<0.01, ns indicates not significant. Bars represent the mean value for each group and error bars are standard error of the mean. Data are pooled from three or more independent experiments.

To test how sensitive Tfh cells were to loss of GC B cells, SRBC-immunized mice were treated with titrated doses (0.4–2 mg/mouse) of anti-CD40L-TM MAb to eliminate GC B cells. The MAb was administered at days 9 and 11 post SRBC immunization, when mature Tfh and GC B cells are stably established (Figure 4A). Treatment with a low dose of anti-CD40L-TM MAb (0.4 mg/mouse) reduced splenic GC B cells by >90% (p<0.001) and resulted in a 25–40% decrease on Tfh cells (Figure 4B–D). In spleen sections from these mice, small GC clusters, although greatly reduced in size, remained in the spleens and were still interacting with Tfh cells suggesting that a few GC B cells were sufficient to maintain several Tfh (data not shown). A higher dose (1 mg per mouse), led to a >99% decrease in GC B cells (∼1000 GC B cells per spleen; p<0.001) resulting in a further decrease in Tfh numbers compared to the 30–60% decrease observed in isotype control treated mice (p<0.01). When GC B cell numbers dropped below 1000 cells per spleen, achieved by treating the mice with the highest dose of 2 mg/mouse, a more complete reduction in Tfh numbers was detected (consistently >60% decrease in Tfh relative to isotype control-treated mice; p<0.001) (Figure 4B–D). In summary, a dramatic reduction was seen in the Tfh population after near complete elimination of GC B cells, supporting the hypothesis that GC B cells support Tfh maintenance.

### Follicular Dendritic cells (FDC) are dispensable for Tfh maintenance

**Figure 5:**
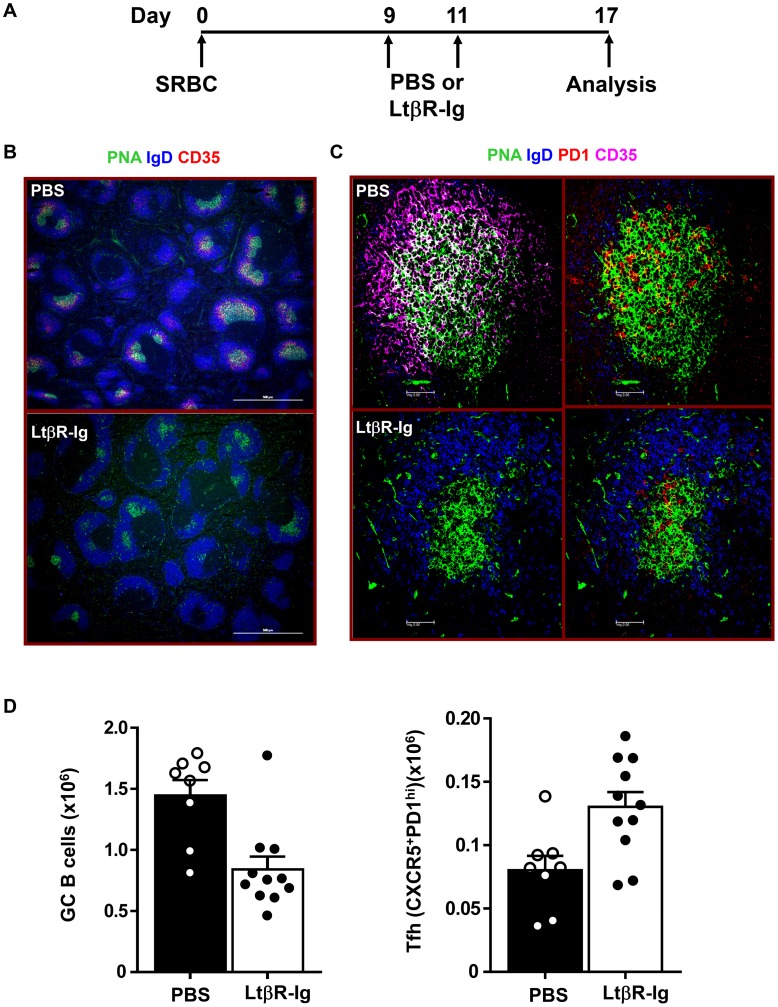
LtβR-Ig treatment in SRBC immunized mice disrupts FDC but does not lower the number of Tfh cells. (A) Diagrammatic representation of experiment protocol. BALB/c mice were immunized with SRBC on day 0 and treated with 0.25 mg of recombinant Lt mg of recombinant LtβR-Ig or PBS on days 9 and 11. Spleens were harvested on day 17 post immunization and subjected to histology and FACS analysis. (B) Cryosections of spleens stained with PNA (green) to detect GC B cells, IgD (blue) to detect follicular B cells and CD35 (red) to detect FDC. Images were captured and analyzed by microscopy. Bar scale represents 500 µm. (C) Sections were stained with PNA (green), PD1 (red) to detect Tfh, IgD (blue) and CD35 (magenta). Bar scale represents 50 µm. (D) The bar graphs show numbers of GC B cells (PNA^+^FAS^high^) and Tfh (CXCR5^+^PD1^high^) cells from either LtβR-Ig or PBS treated mice. Data are pooled from at least eight mice analyzed in two experiments and each dot represents an individual mouse. Bars represent the mean value for each group and error bars are standard error of the mean.

The results described above clearly demonstrate that GC B cells are required for the maintenance of Tfh in splenic follicles. However, GC B cells as well as Tfh cells also interact with follicular dendritic cells (FDC) within the GC structures. To assess the role of these cells in GC maintenance we depleted FDC, by inhibiting lymphotoxin α1β2 signals [49]. BALB/c mice were immunized with SRBC i.p. on day 0, and were randomly separated into two groups, receiving LTβR-Ig treatment at 0.25 mg/mouse or PBS on days 9 and 11. Spleens from all mice were recovered on day 17 for FACS and histological analysis (Figure 5A). There was no detectable staining of the FDC markers CD35, CD157, and Madcam1 inside or around GC in LTβR-Ig treated animals (Figure 5B and Figure S4). In addition, structures with high IgM or C4 signals were not visible in the LTβR-Ig treated spleens (Figure S4C and D), suggesting a complete removal of FDC and their associated immune complexes. However, despite effective FDC depletion following LTβR-Ig treatment, we found that spleen structures were largely intact and PNA^+^ GC B cell clusters though smaller, were clearly present inside B cell follicles (Figure 5).

Immunohistological examination revealed the presence of PNA^+^ GC B cells which colocalized with PD1^high^ Tfh cells (Figure 5C). We further quantified the numbers of Tfh and GC B cells by flow cytometry, and although LTβR-Ig treatment decreased GC B cells numbers by 50%, the numbers of Tfh cells in LTβR-Ig treated mice were instead increased by ∼45–60% compared to mice receiving PBS (Figure 5D). Therefore we concluded that FDC are not required for supporting Tfh cells during an ongoing GC reaction and LTβR-Ig treatment may in fact play a role in Tfh expansion and/or survival.

### ICOS-ICOSL but not CD28-B7 signaling is required for maintenance of Tfh cells

Coordinated interaction between the costimulatory molecules CD28 and ICOS and their ligands on DC and B cells are crucial for Tfh differentiation [4], [33]. Mature GC B cells are capable of influencing Tfh cells through both pathways because they constitutively express both B7.2 and ICOS ligand [13], [61]. Here we investigated the involvement of these pathways in supporting the maintenance of mature Tfh cells by using antagonist reagents CTLA4-Ig and anti-ICOS-TM MAb in the SRBC immunization model.

First we confirmed that CTLA4-Ig and anti-ICOS-TM MAb have blockade function as described previously [72], [73]. Treatment with CTLA4-Ig or anti-ICOS-TM MAb, shortly before and after immunization with SRBC (on days −1, 1 and 3), resulted in dramatically lower Tfh numbers (>85% lower) on day 7 post immunization (Figure S3E–H and data not shown). Pre- and early treatment with these MAbs effectively abrogated Tfh differentiation as described in the literature and confirmed that these reagents could effectively block the respective pathways as previously described [4].

**Figure 6:**
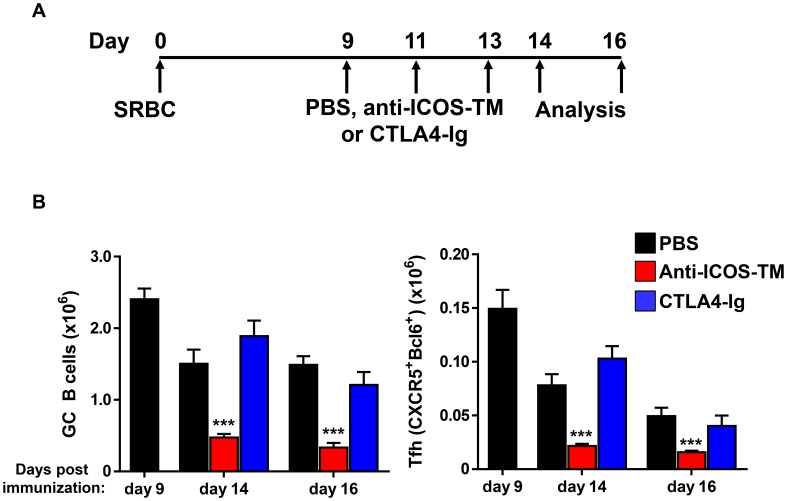
Blocking ICOS but not CD28 signaling results in decreased Tfh in the maintenance phase of the SRBC immunization model. (A) BALB/c mice were immunized with SRBC on day 0 and treated with 0.5 mg of recombinant CTLA4 mg of recombinant CTLA4-Ig, anti-ICOS-TM MAb or isotype control MAbs on days 9, 11 and 13. Spleens were harvested on day 14 and 16 post immunization and subjected to flow cytometry analysis. (B) Graphs show the numbers of GC B cells (PNA^+^FAS^high^IgD^low^) (A) and Tfh cells (CXCR5^+^Bcl-6^+^) over time in mice treated with MAbs or PBS. Cell numbers on day 9 were pooled form untreated BALB/c mice receiving the same immunization. Cell numbers on day 14 and 16 from CTLA-4 Ig or anti-ICOS-TM MAb treated mice were compared to mice dosed with PBS. Data are pooled from two independent experiments with n = 4∼8 per group. Error bars are standard error of the mean. Numbers of GC B cells and Tfh cells in anti-ICOS-TM treated mice on day 14 and 16 were significantly lower than those in PBS treated mice (***, p<0.001).

Next, CD28 and ICOS pathways were blocked during an ongoing immune response (Day 9, 11, 13 post SRBC immunization) by treatment with CTLA4-Ig or anti-ICOS-TM MAb. The number of Tfh cells and GC B cells in mice treated with CTLA4-Ig (0.5 mg/mouse x3) were similar to the number from mice treated with control reagent (Figure 6A–B). Thus, while CD28-associated pathway was required for Tfh differentiation, it is dispensable for the maintenance of Tfh cells during ongoing immune response to SRBC [4], [74]. In contrast to the result after CTLA-4 Ig treatment, blocking ICOS-ICOSL signaling in the maintenance phase of SRBC-induced GC responses led to an ∼80% reduction of GC B cells (p<0.001) and concomitant ∼70% reduction of Tfh cells compared to isotype control or PBS treated mice (Figure 6A–B). These effects of ICOS blockade on Tfh maintenance were consistent with a previously published study with ovalbumin immunization) [40]. In order to further rule out that the effects of anti-ICOS-TM MAb was not due to direct depletion of Tfh cells, we first engineered an anti-ICOS MAb with a murine IgG1 Fc. IgG1 Fc has minimal effector functions mainly through FcγRIII [48]. Mice deficient in FcγRIII were treated with our IgG1 Fc engineered anti-ICOS MAb at day 9 post SRBC immunization. A similar reduction in Tfh and GC B cells after anti-ICOS mouse IgG1 treatment was observed in this model (data not shown). Taken together, these data suggest that ICOS-ICOSL signaling is critical for Tfh maintenance whereas CD28 signaling, although required for Tfh development, does not appear to play a role in the maintenance phase in this model.

**Figure 7:**
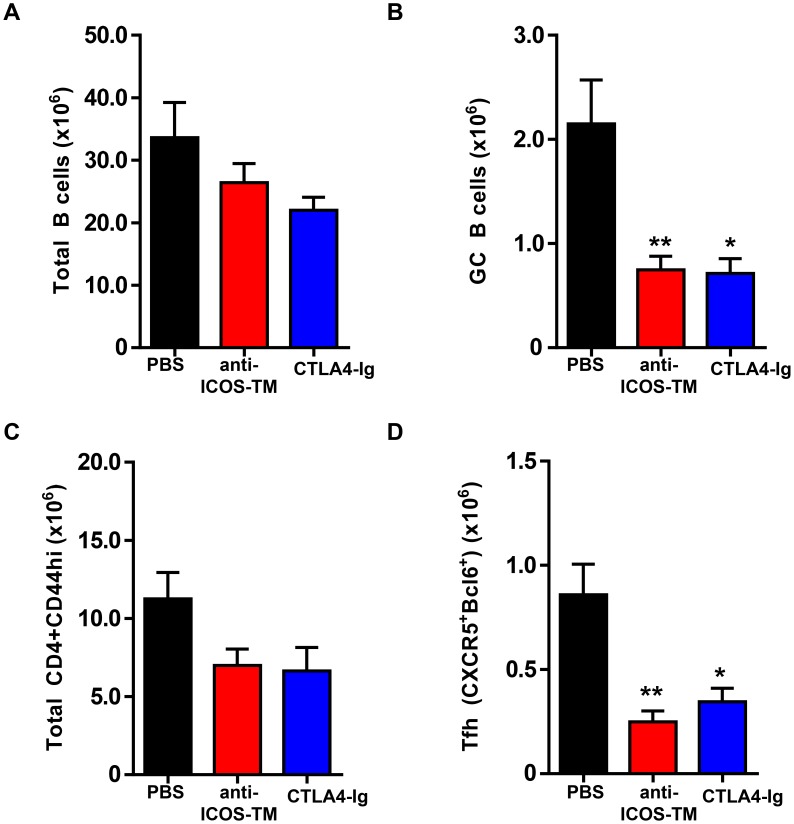
Maintenance of Tfh cells in NZB/W F1 mice requires ICOS signaling. Female NZB/WF1 mice at 6 months of age were treated with CTLA4-Ig (1 mg mg/mouse) anti-ICOS-TM (1 mg mg/mouse), or PBS at days 0, 2 and 4. Mice were sacrificed and splenocytes were analyzed by FACS at day 7 post first antibody dosing. (A–D) Graphs show number of total B cells (B220^+^CD19^+^) in (A), GC B cells (PNA^+^FAS^high^IgD^low^) in (B), Total CD44^high^ cells in (C) and Tfh cells (CXCR5^+^PD1^high^) in (D). **, P<0.01 *, P<0.05. Data are representative of three independent experiments. Bars represent the mean value for each group and error bars are standard error of the mean.

We next investigated whether these two signaling pathways would be important for maintenance of autoreactive Tfh. NZB/W F1 mice between the ages of 5 to 6 months were examined, a time when spontaneous GC B cells and Tfh cells are present in all NZB/W F1 mice from our colony (Figure S1B). These mice were treated with CTLA4-Ig or anti-ICOS-TM MAb on days 0, 2, and 4 and splenocytes were analyzed on day 7. Similar to what was observed in the SRBC-immunized BALB/c mice, blocking ICOS signaling led to a significant decrease in number of both GC B cells (∼65% reduction, p<0.01) and Tfh (CD4^+^ CD44^high^ CXCR5^+^Bcl6^+^) cells (∼70% reduction, p<0.01) in this spontaneous autoimmune model of lupus (Figure 7 B and D). These effects were specific to GC B cells and Tfh cells as the frequencies of total B cells and total effector/memory (CD4^+^ CD44^high^) T cells were unaffected after anti-ICOS-TM MAb treatment (Figure 7 A and C). Interestingly, CTLA4-Ig treatment in this spontaneous model also reduced GC B cell numbers significantly (∼65% reduction, p<0.05). This reduction was comparable to the effects of blocking the ICOS pathway. Furthermore, as expected by the reduction in GC B cells, CTLA-4 Ig treatment also led to a significant decrease in Tfh (Figure 7B and D). These data suggest that in both immunization and autoimmune settings, ICOS-ICOSL signals play a critical role in the maintenance of Tfh cells. In contrast, the maintenance autoreactive Tfh is dependent on the CD28 pathway while being dispensable for Tfh generated by immunization with an exogenous antigen.

## Discussion

Most pathogenic auto-antibodies show evidence of somatic hypermutation (SHM) strongly suggesting that they arise from GC responses [1], [7]–[9]. Additionally, the presence of spontaneous GC B cells has been documented in many of the existing murine disease models and in ectopic follicles from patients with autoimmune disease [1], [7]–[9], [16], [17], [31]. Taken together, these data support an important role for the GC reaction in autoimmune diseases. Dissecting the relationship among the cell types present in the GC microenvironment is crucial for a better understanding of mechanisms underlying current treatments and can lead to the development of novel therapeutics. In clinical studies B cell depletion therapy has shown efficacy in multiple autoimmune diseases [75]. Depending on disease pathophysiology and patient subset, several aspects of B cell depletion may contribute to the observed clinical effect. These include reduction of antigen presentation and secretion of inflammatory cytokines by B cells as well as the potential to reduce serum titers of some autoantibodies [75]. Our results suggest an additional mechanism, where depletion of GC B cells results in elimination of mature Tfh cells, which are the main driver for GC reaction in autoimmune diseases and are also a source of pro-inflammatory cytokines, such as IL-21 [4].

Increased numbers of Tfh cells are found in many autoimmune mouse models, providing an opportunity to investigate the mechanisms and pathways required for their differentiation as well as their maintenance, the focus of the present report [2], [3], [15]–[19]. A previous report suggested the GC B cells are important for Tfh maintenance in protein immunization models [40]. In autoimmune models, earlier studies suggested that certain cytokines produced by Tfh cells are important not only for Tfh function but also for their maintenance. For example, Tfh cells in the sanroque model produce IFN-γ and blockade of IFN-γ function can reduce the number of Tfh cells [76]. In autoimmune BXD2 mice Tfh cells make IL-17 and blockade of IL-17 led to Tfh cells moving out of GC [77]. Here, we targeted reciprocal interactions between GC B cells and Tfh cell based on the observation that Tfh cells in autoimmune mice concentrate inside GC, making direct contact with GC cells (Figure 1B). As demonstrated in the current study, elimination of GC B cells has the potential to reduce autoreactive Tfh cells, through a mechanism which is independent of cytokines produced by Tfh cells.

Depletion of total B cells in the Sle1.hCD19Tg mice led to a drastic reduction of Tfh cell numbers within a week of a single intravenous dose of the high-affinity anti-CD19 MAb, MEDI-551. This rapid effect was specific to the Tfh subset with no significant changes seen in the general CD44^high^ CD4 T cell memory and effector population (Figure 1). Our results demonstrate that B cell depletion in mice with an established autoimmune phenotype leads to a rapid loss of mature Tfh cells, highlighting a continued interplay between these two cell types even in an autoreactive environment where tolerance is lost.

We observed in autoimmune and SRBC immunized mice, that substantial reductionof GC B cells is required to induce significant decrease in Tfh cells. It has been reported that GC B cells, compared to other B cell subsets are relatively resistant to anti-CD20 immunotherapy in human-CD20 transgenic mice [40], [78] (and Figure S3B). Therefore, the depleting MAb itself needs to be optimized in order to achieve maximum depletion of GC B cells. One approach is to increase MAb depleting potency through engineering. For example, MEDI-551 is an affinity optimized and carbohydrate-engineered MAb lacking fucose in the Fc region, which results in enhanced potency in B cell depletion in multiple mouse models ([46] and data not shown). Alternatively, as shown in this study, a combination immunotherapy of anti-CD20 plus anti-CD40L or anti-CD40L alone can be utilized to effectively eliminate both naïve and GC B cells or GC B cells respectively, resulting in significant reduction of the number of Tfh cells (Figures 2 and 3).

A recent study which focused on immunization models, reported a requirement for antigen and GC B cells, through ICOS signaling, in sustaining the Tfh phenotype after protein immunization [40]. However, factors required for Tfh maintenance in an autoimmune setting where tolerance has been lost have not been explored previously. Using anti-CD40L-TM blocking MAb to specifically disrupt GC B cells in both NZB/W F1 and SRBC immunized mice, we found that GC B cells in particular control the maintenance of mature Tfh cells in a spontaneous autoimmune model. Furthermore, depletion of non-GC B cells did not reduce Tfh numbers (Figures 2, 4 and Figure S3B). We now provide experimental evidence that direct contact between GC B cells and Tfh cells is important for the maintenance of autoreactive Tfh cells in two well-established models of autoimmunity, Sle1 and NZB/W F1 mice.

The role of FDC in supporting GC B cells and Tfh cells was previously studied using CD21-DTR mice in which FDC are depleted following diphtheria toxin (DTx) treatment [79]. After immunization with SRBC for 6-8 days, mice were treated with DTx to deplete FDCs, resulting in grossly altered lymphoid structure in the spleen and a loss of GC B cell clusters, accompanied by 6- and 2-fold reduction of splenic GC B cells and Tfh cells, respectively. In our studies, although treatment of LtβR-Ig in SRBC immunized mice almost completely eliminated splenic FDC structures, GC B cells remained as clearly formed clusters within follicles (Figure 5). The differential consequence on GC clustering after FDC ablation in the two studies are likely a result of the different model systems: FDC ablation by DTx treatment was fast and completed by 2 days. In comparison, loss of FDC after LtβR-Ig took 7–8 days. Using LTb^−/−^ bone marrow chimeras to generate mice lacking FDCs, a recent study arrived at a conclusion similar to our results suggesting that FDCs are not required for GC B cells. This study however, did not look at the effects of FDC depletion on Tfh [80]. Interestingly, DTx treatment did not abolish CXCL13 mRNA expression or sphingosine-1-phosphate (S1P) gradient, indicating a FDC-independent mechanism for controlling B cell migration [79]. We suspect CXCL13 and/or S1P pathways may have compensated for the slow loss of FDC in the LtβR-Ig treated mice, which was sufficient to support partial GC clustering, and thereby support Tfh cells. The current study with LTβR-Ig treatment did not rule out a contribution of FDC for Tfh migration and positioning in the first week of GC responses, and again confirmed an important role of FDC in supporting optimal numbers of GC B cells. However it appears that in established GC, signals supporting mature Tfh cells are exclusively provided by GC B cells.

**Figure 8:**
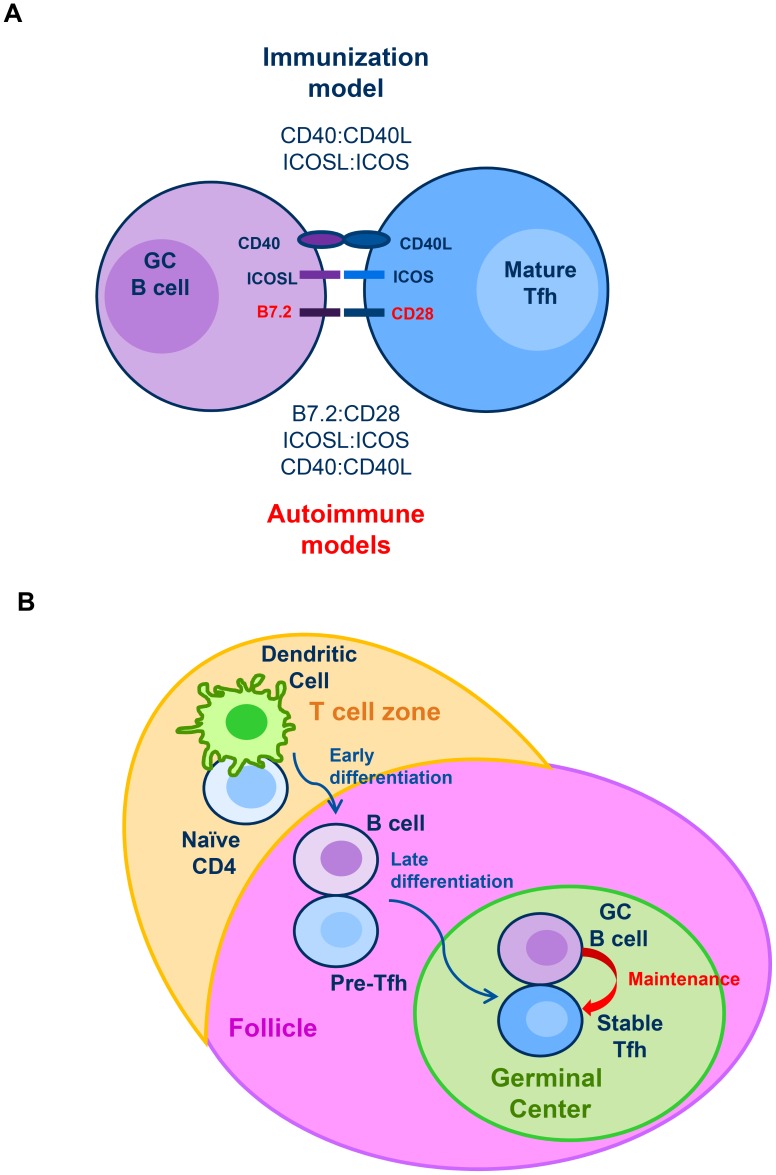
Model of Tfh differentiation and maintenance. (A) Our data suggest that in both immunization and autoimmune mouse models, GC B cells are actively providing signals to maintain optimal Tfh numbers during the course of the GC reaction. In the immunization model, ICOSL:ICOS but not B7.2:CD28 signaling, is required for Tfh maintenance. Interestingly, in the spontaneous autoimmune model both signals are required to maintain optimal numbers of mature Tfh. (B) Previous studies have shown that Tfh differentiation is a multistage process: Dendritic cells initiate the differentiation of naïve T cells into pre-Tfh. Upon upregulation of CXCR5, these pre-Tfh are able to enter the follicle and interact with cognate B cells. This interaction with B cells drives the full polarization of Tfh into mature Tfh which is required for the germinal center reaction. Our data in an immunization model demonstrate that once fully mature, Tfh continue to require signals from GC B cells to sustain their maintenance. We further show that the same B cell dependence continues to play a role in spontaneous models of autoimmunity.

Since B cells express ligands for ICOS and CD28 expressed on Tfh cells, we first tested the requirement for these pathways in the maintenance of Tfh in SRBC immunized mice. In addition to its role in Tfh differentiation and consistent with a previous study using protein immunization, we confirmed an important role for ICOS in Tfh maintenance. Intriguingly however, blocking CD28 signaling with CTLA4-Ig did not affect the numbers of either GC B cells or Tfh in this model. The lacks of an effect of CD28 blockade on GC B cells is consistent with an earlier study which did not specifically look at Tfh cells [74]. Our observations on Tfh demonstrate demonstrate that although the CD28 pathway is mandatory for optimal Tfh differentiation, this pathway plays a marginal role for Tfh maintenance, which highlights a fundamental signaling difference between Tfh differentiation and maintenance (Figure 6) [4], [33]. We next explored whether these two costimulatory pathways are essential for sustaining Tfh cells in autoimmune NZB/W F1 mice. Interestingly, in this spontaneous model of autoimmunity, both pathways were required to maintain optimal levels of Tfh, highlighting a crucial difference in the two models (Figure 7 and 8). Previous studies have found that B cells can also influence Tfh function and development through pathways such as PD1 and SLAM receptors, and while their role in maintenance of autoreactive Tfh requires further investigation [4], [81]–[83], our data clearly illustrate non-redundant roles for ICOS and CD28 in the maintenance phase in autoimmune mice. The fate of effector Tfh after depletion of GC B cell or blocking ICOS and CD28 pathways will also require further studies. However, it is unlikely that effector Tfh cells are able to differentiate into memory Tfh cells in the absence of GC B cells because prior studies demonstrated the formation of memory CD4 T cells depends on signals provided by B cells and ICOS [84]–[86].

Elevated numbers of Tfh cells have not only been reported in mouse models of autoimmunity [2], but several studies have now demonstrated expansion of Tfh cells in the peripheral blood of patients with a variety of autoimmune diseases including SLE [3], [20], [30], [40], [87]. One such study showed that these circulating Tfh cells express CXCR5 and IL-21, similar to the Tfh cells observed in the spleens from autoimmune mice [87]. Therefore, excessive Tfh cells may drive the development of autoimmunity in both humans and mice. However mechanisms that control the persistence of Tfh cell in SLE patients are currently unknown. We showed here in autoimmune mouse models that GC B cells and costimulatory pathways, ICOS and CD28, control the maintenance of established Tfh. By focusing on time-points when spontaneous GC B cells and Tfh were already present at elevated levels, our data demonstrate that these treatments will disrupt the maintenance of existing mature Tfh (Figure 8).

In summary, by demonstrating the dependence of fully mature Tfh on GC B cells in sustaining their maintenance, our study highlights the significance of crosstalk between GC B cells and Tfh cells in regulating both immunization induced humoral immune responses as wells as spontaneous autoimmune responses. Our results suggest that targeting Tfh or GC B cells, which are reciprocally dependent on each other, may potentially eliminate both cell types. It provides further rationale for targeting B cells and costimulatory pathways, such as ICOS and CD28, in autoimmune diseases by highlighting their potential to dampen autoreactive responses by eliminating both GC B cells and Tfh cells. In fact, depletion of B cells with rituximab in type 1 diabetes patients showed a reduction of circulating Tfh like cells [88]. Tracking the changes of the circulating T helper cells in clinical studies will provide further insight into the interplay between B cells and Tfh cells in the context of human disease.

## Supporting Information

Figure S1 **Number of GC B cells and Tfh cells in autoimmune mouse models.** (A) Number of GC B cells (B220^+^CD19^+^PNA^+^FAS^high^IgD^low^) and Tfh cells (CD4^+^B220^−^CD44^high^CXCR5^+^PD1^high^) in B6, hCD19Tg, Sle1 and Sle1-hCD19 Tg mice, of the indicated age. Dots display the mean and error bars indicate standard error of mean (SEM). N> = 4–23 per group (***, *p*<0.001 B6 vs. Sle1 and hCD19 Tg vs. Sle1-hCD19 Tg at last timepoint). (B) Spleens were harvested from 2.5 to 3.5-month C57BL/6 (B6) mice either unimmunized or 14 days post SRBC immunization. Numbers of GC B cells (B220+CD19+PNA+FAShighIgDlow) and Tfh cells (CD4+CXCR5+PD1high) were enumerated by flow cytometry analysis and plotted in the graph. Each dot represents a single mouse of indicated treatment group. (C) Bar graphs show number of GC B cells and Tfh cells in NZB/W F1 mice at 3 and 5 months of age. Each symbol represents one mouse. Dots display average and error bars indicate standard error of mean (SEM). N = 4 per group. * *p*<0.05 with Student's t test. (D) Histograms showing Bcl6, Foxp3, GL-7, and Ki67 expression between CXCR5-PD1low and CXCR5+PD1high CD4+ T cells from 9 to 12 month old Sle1-hCD19 Tg mice. Error bars indicate standard error of mean (SEM). N = 4 per group. * p<0.05 with Student's t test.
(TIF)

Figure S2 **Kinetics of GC B cells and Tfh cells in BALB/c mice immunized with SRBC.** BALB/c mice were immunized with SRBC and spleens cells were collected and analyzed at day 7, 9, 14, 21 and 30 post immunization. (A) Means of GC B cells (B220^+^CD19^+^PNA^+^FAS^high^IgD^low^) and Tfh (CD4^+^B220^−^CD44^hi^CXC5+PD1^high^). (B) Means of GC B cells (Bcl-6^+^) and Tfh cells (CXCR5^+^Bcl-6^+^ cells). (C) Means of Tfh cells: GL7^+^Tfh (GL7^+^SLAM^lo^) cells, Ki67^+^ Tfh cells, and Foxp3^+^ TFR cells (CXCR5^+^Bcl-6^+^Foxp3^+^). (D) Histological sections of spleens from SRBC immunized mice. Sections show staining for GC B cells (PNA^+^, blue), Tfh cells (CD4^+^PD1^+^, yellow), and CD4^+^ T cell zone (green). Data are representative of two independent experiments.
(TIF)

Figure S3 **Treatment with anti-CD20 MAb and CTLA4-Ig in SRBC immunized BALB/c mice.** (A) A schematic view of SRBC immunization and anti-CD20 treatment protocol. A cohort of BALB/c naïve mice were immunized with SRBC at day 0 and were treated at day 9 with 0.25 mg/mouse of anti-CD20 MAb or PBS. Spleens were recovered at Day 17 and analyzed by FACS. (B) B cells numbers (B220^+^murine CD19^+^), (C) GC B cell numbers (PNA^+^Fas^+^) and (D) Tfh (CXCR5^+^PD1^high^) numbers per spleen at Day 17. Graphs show the means and standard deviation of mean. N = 5 per group. Significant differences (***, *p*<0.001) were between anti-CD20 MAb and PBS group. (E) A schematic view of SRBC immunization and CTLA4-Ig treatment protocol. A cohort of naïve BALB/c mice were immunized with SRBC at day 0 and treated at days −1, 1 and 3 with 0.4 mg/mouse of CTLA4-Ig or PBS. Spleens from treated mice were recovered on day 7 and analyzed with FACS. (F–H) Bar graphs show numbers of total B cells (B220^+^CD19^+^) per spleen in (F), GC B cells (PNA^+^FAS^high^IgD^low^) per spleen (G) the numbers of Tfh cells (CXCR5^+^PD1^high^) (H) gated on CD4+CD44^high^ T cells per spleen. *** *p*<0.001. N = 4 per group. Bars represent the mean value for each group and error bars are standard error of the mean.
(TIF)

Figure S4 **LtβR-Ig treatment in SRBC immunized mice disrupts FDCs.** Mice were immunized with SRBC and treated as shown in Figure 5. (A–D) Cryosection of spleens from LtβR-Ig or PBS treated mice were stained with PNA (green), anti-IgD (blue) and anti-CD157 (red) in (A), PNA (green), anti-IgD (blue) and anti-Madcam1 (red) in (B), PNA (green) and anti-IgM Fcμ chain (red) in (C) and PNA (green) and C4 (red) in (D). Images were captured and analyzed by microscopy. Bar scale represents 500 µm.
(TIF)
