# Supplementary figures and images for: Germinal Center B Cell Depletion Diminishes CD4+ Follicular T Helper Cells in Autoimmune Mice
Source: PLoS One. 2014 Aug 7;9(8):e102791. doi: 10.1371/journal.pone.0102791 (PMC4125140; doi:10.1371/journal.pone.0102791)

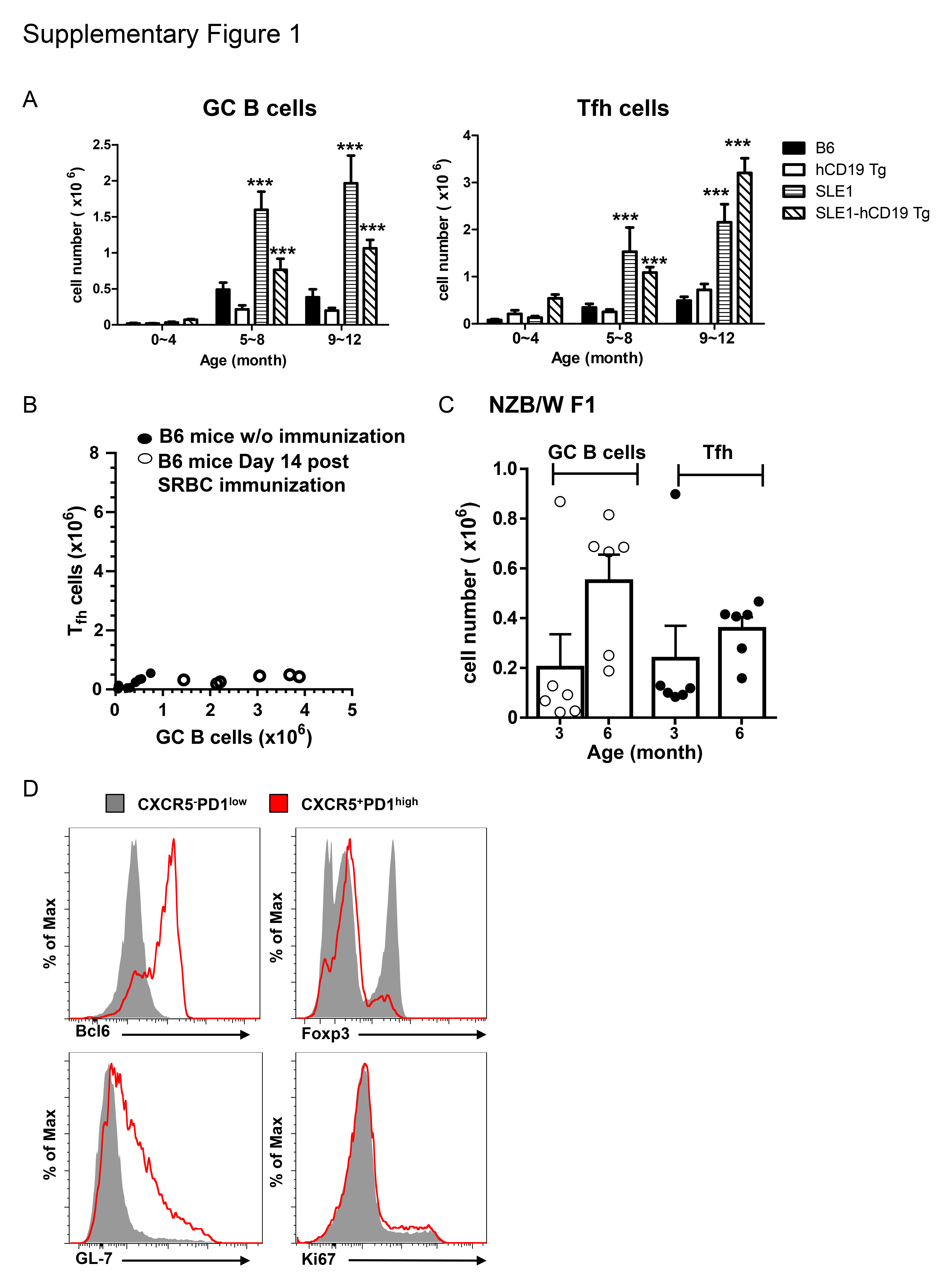

Supplement: Figure S1 — Number of GC B cells and Tfh cells in autoimmune mouse models. (A) Number of GC B cells (B220+CD19+PNA+FAShighIgDlow) and Tfh cells (CD4+B220−CD44highCXCR5+PD1high) in B6, hCD19Tg, Sle1 and Sle1-hCD19 Tg mice, of the indicated age. Dots display the mean and error bars indicate standard error of mean (SEM). N> = 4–23 per group (***, p<0.001 B6 vs. Sle1 and hCD19 Tg vs. Sle1-hCD19 Tg at last timepoint). (B) Spleens were harvested from 2.5 to 3.5-month C57BL/6 (B6) mice either unimmunized or 14 days post SRBC immunization. Numbers of GC B cells (B220+CD19+PNA+FAShighIgDlow) and Tfh cells (CD4+CXCR5+PD1high) were enumerated by flow cytometry analysis and plotted in the graph. Each dot represents a single mouse of indicated treatment group. (C) Bar graphs show number of GC B cells and Tfh cells in NZB/W F1 mice at 3 and 5 months of age. Each symbol represents one mouse. Dots display average and error bars indicate standard error of mean (SEM). N = 4 per group. * p<0.05 with Student's t test. (D) Histograms showing Bcl6, Foxp3, GL-7, and Ki67 expression between CXCR5-PD1low and CXCR5+PD1high CD4+ T cells from 9 to 12 month old Sle1-hCD19 Tg mice. Error bars indicate standard error of mean (SEM). N = 4 per group. * p<0.05 with Student's t test. (TIF) [file pone.0102791.s001.tif]

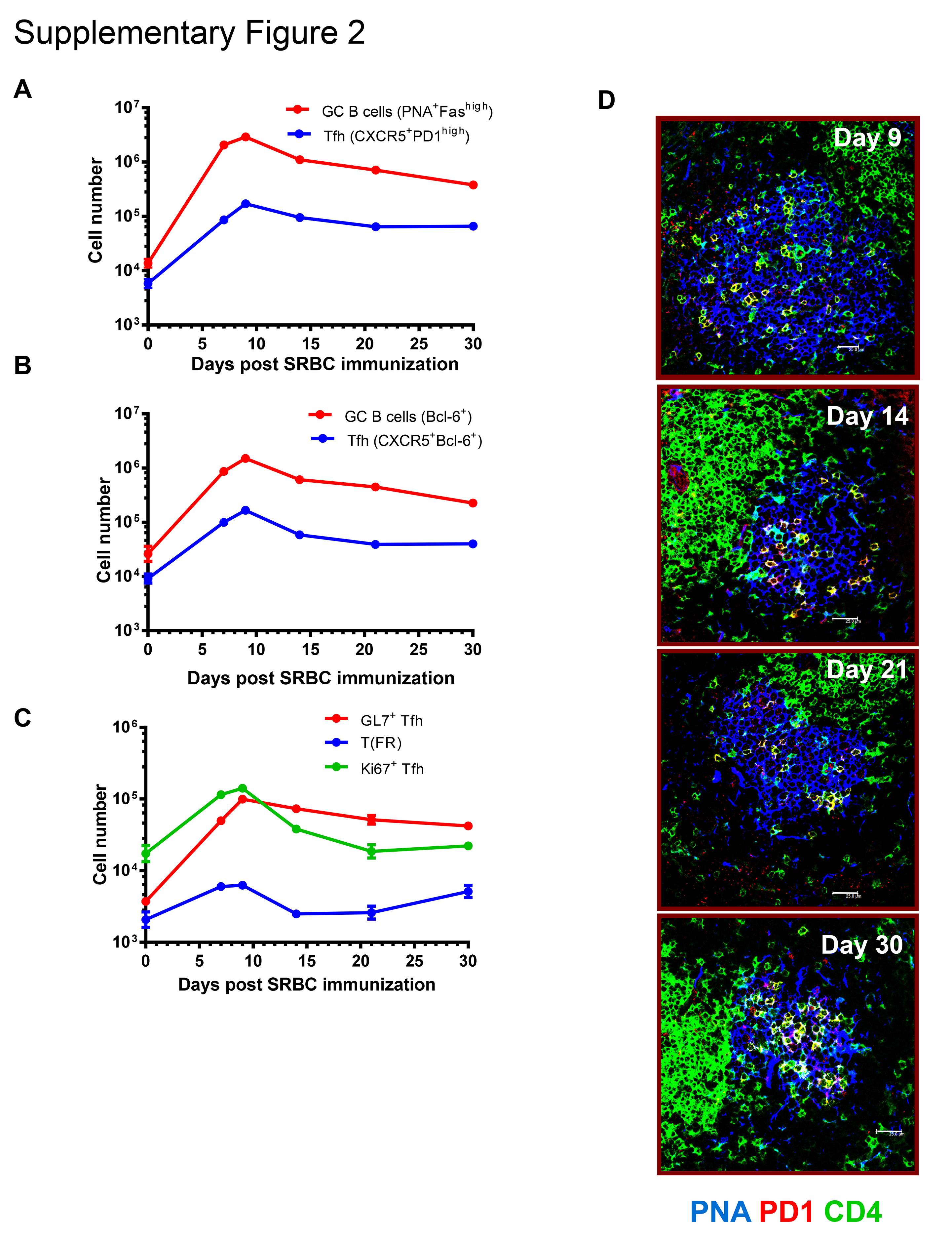

Supplement: Figure S2 — Kinetics of GC B cells and Tfh cells in BALB/c mice immunized with SRBC. BALB/c mice were immunized with SRBC and spleens cells were collected and analyzed at day 7, 9, 14, 21 and 30 post immunization. (A) Means of GC B cells (B220+CD19+PNA+FAShighIgDlow) and Tfh (CD4+B220−CD44hiCXC5+PD1high). (B) Means of GC B cells (Bcl-6+) and Tfh cells (CXCR5+Bcl-6+ cells). (C) Means of Tfh cells: GL7+Tfh (GL7+SLAMlo) cells, Ki67+ Tfh cells, and Foxp3+ TFR cells (CXCR5+Bcl-6+Foxp3+). (D) Histological sections of spleens from SRBC immunized mice. Sections show staining for GC B cells (PNA+, blue), Tfh cells (CD4+PD1+, yellow), and CD4+ T cell zone (green). Data are representative of two independent experiments. (TIF) [file pone.0102791.s002.tif]

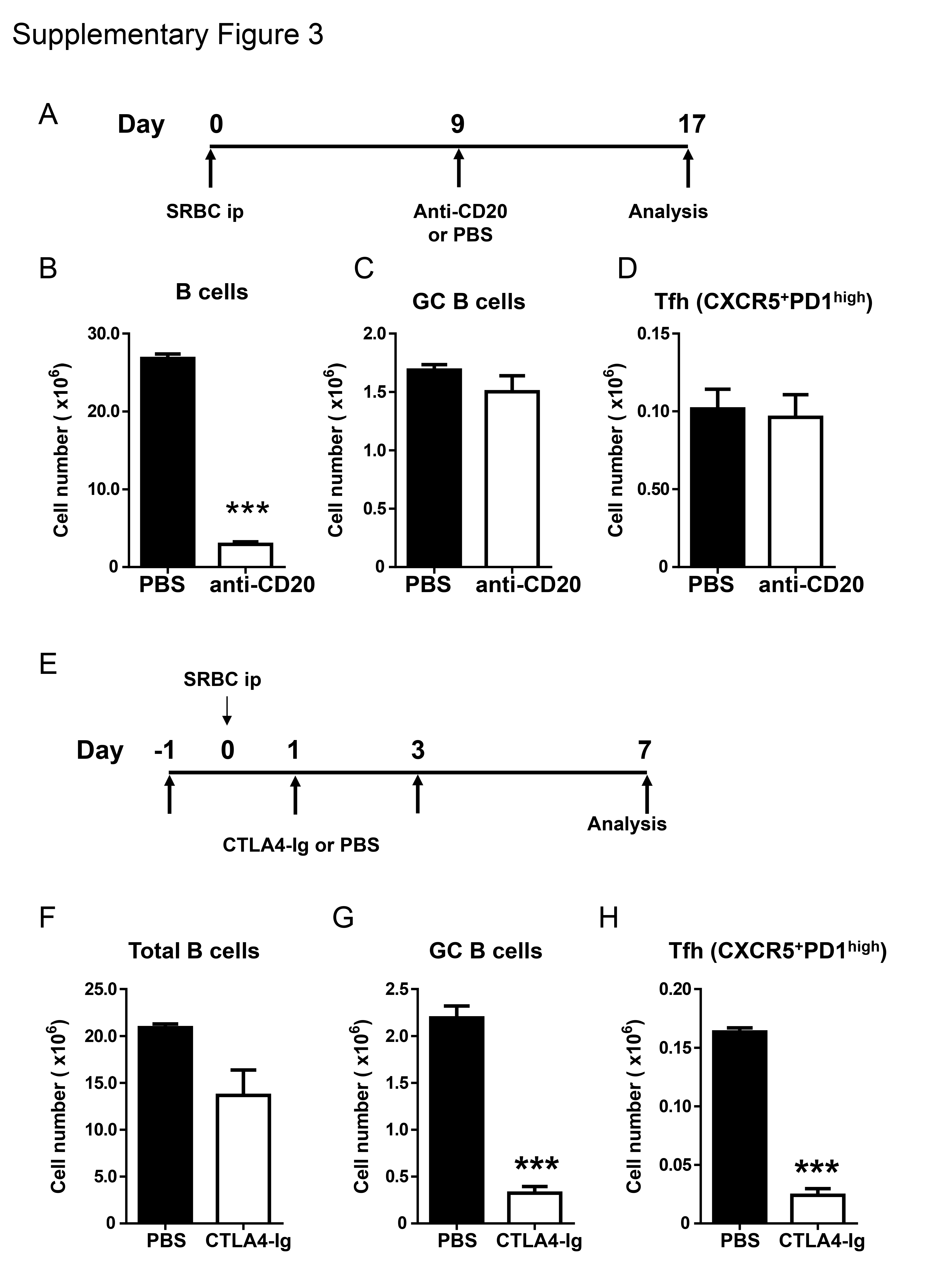

Supplement: Figure S3 — Treatment with anti-CD20 MAb and CTLA4-Ig in SRBC immunized BALB/c mice. (A) A schematic view of SRBC immunization and anti-CD20 treatment protocol. A cohort of BALB/c naïve mice were immunized with SRBC at day 0 and were treated at day 9 with 0.25 mg/mouse of anti-CD20 MAb or PBS. Spleens were recovered at Day 17 and analyzed by FACS. (B) B cells numbers (B220+murine CD19+), (C) GC B cell numbers (PNA+Fas+) and (D) Tfh (CXCR5+PD1high) numbers per spleen at Day 17. Graphs show the means and standard deviation of mean. N = 5 per group. Significant differences (***, p<0.001) were between anti-CD20 MAb and PBS group. (E) A schematic view of SRBC immunization and CTLA4-Ig treatment protocol. A cohort of naïve BALB/c mice were immunized with SRBC at day 0 and treated at days −1, 1 and 3 with 0.4 mg/mouse of CTLA4-Ig or PBS. Spleens from treated mice were recovered on day 7 and analyzed with FACS. (F–H) Bar graphs show numbers of total B cells (B220+CD19+) per spleen in (F), GC B cells (PNA+FAShighIgDlow) per spleen (G) the numbers of Tfh cells (CXCR5+PD1high) (H) gated on CD4+CD44high T cells per spleen. *** p<0.001. N = 4 per group. Bars represent the mean value for each group and error bars are standard error of the mean. (TIF) [file pone.0102791.s003.tif]

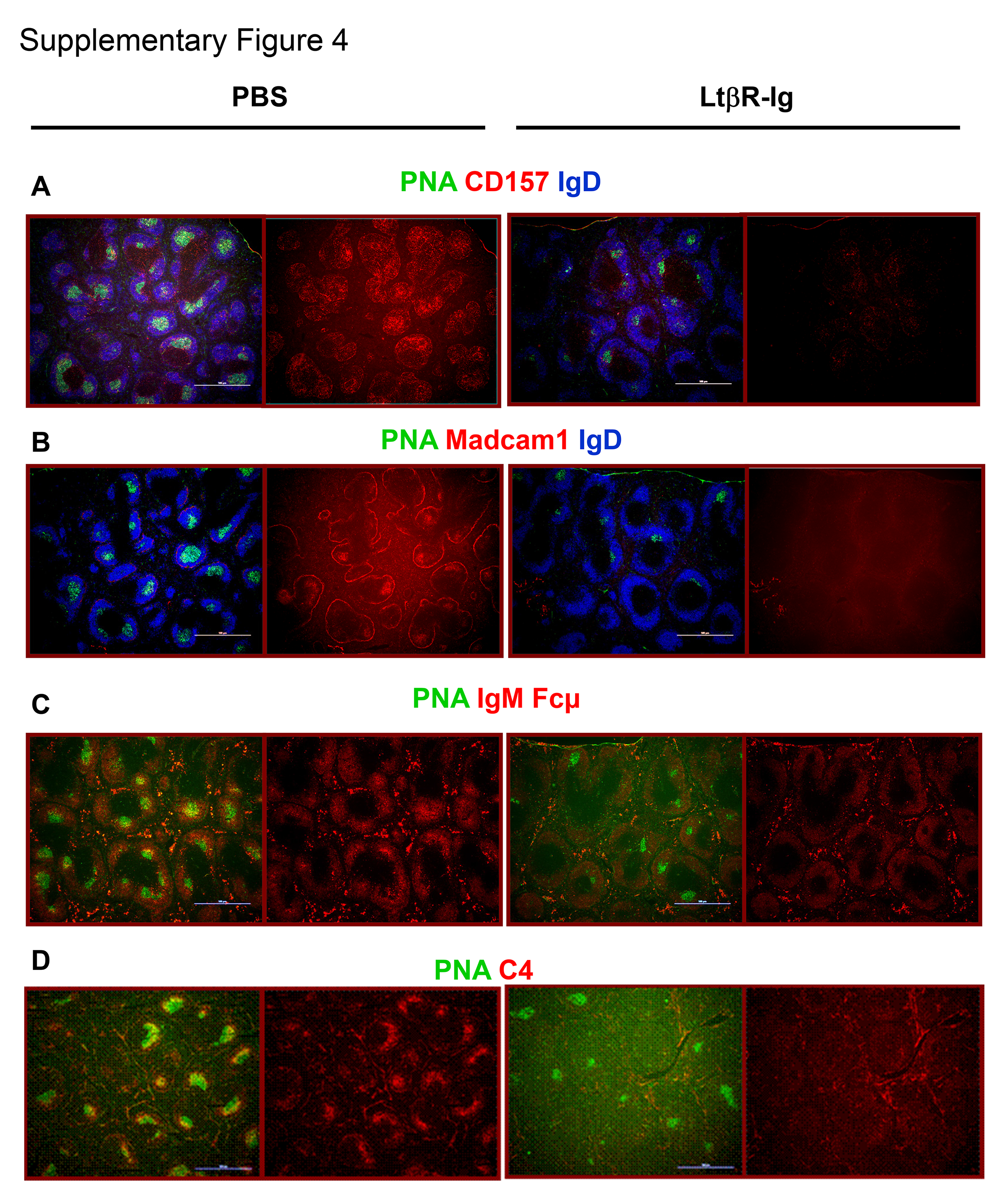

Supplement: Figure S4 — LtβR-Ig treatment in SRBC immunized mice disrupts FDCs. Mice were immunized with SRBC and treated as shown in Figure 5. (A–D) Cryosection of spleens from LtβR-Ig or PBS treated mice were stained with PNA (green), anti-IgD (blue) and anti-CD157 (red) in (A), PNA (green), anti-IgD (blue) and anti-Madcam1 (red) in (B), PNA (green) and anti-IgM Fcμ chain (red) in (C) and PNA (green) and C4 (red) in (D). Images were captured and analyzed by microscopy. Bar scale represents 500 µm. (TIF) [file pone.0102791.s004.tif]
